# Parental grief and recovery after the stillbirth: An interview study with parents in Sweden one year after stillbirth

**DOI:** 10.18332/ejm/219001

**Published:** 2026-03-31

**Authors:** Berit Höglund, Ingegerd Hildingsson

**Affiliations:** 1 Department of Women’s and Children’s Health, Uppsala University, Uppsala, Sweden; 2 Department of Nursing, Mid Sweden University, Sundsvall, Sweden

**Keywords:** bereaved parents, indelible memories, longitudinal study, recovery after stillbirth, quality of life

## Abstract

**INTRODUCTION:**

Few parents face stillbirth in Sweden, and knowledge of their long-term grieving and recovery process is limited. The aim of the study was to explore parents’ memories, perspectives, reflections, and insights regarding the grieving and recovery process one year after stillbirth.

**METHODS:**

This is a mixed-methods study involving nine in-depth interviews and responses to eleven quantitative statements in convergent succession. Data were analyzed using descriptive statistics and thematic network analysis.

**RESULTS:**

Two themes were identified: 1) ‘Impossible to close the heavy door to indelible memories of the stillborn baby’, and 2) ‘The balance of personal and social factors and internal processes enables increased quality of life’. These themes captured parents’ individual and ongoing processes of grieving and reflection. One year later, parents affirmed increasing stability in their memories and feelings, although more than half described their mental health as partly good/not good. Some parents disclosed new pregnancies and live births, which, per se, called for increased, structured, safe, and respectful professional healthcare that acknowledges previous experiences of stillbirth. The quantitative statements reinforced qualitative findings and showed that parents usually responded positively to holding the baby, and highlighted the importance of knowing the reason behind the stillbirth.

**CONCLUSIONS:**

Bereaved parents experience individual recovery processes, characterized by gradual improvements in quality of life while maintaining a sense of loss for their stillborn baby, one year after the event.

## INTRODUCTION

Stillbirth is a rare outcome in Sweden, occurring at a rate of 3.0 per 1000 live births annually, compared with a global rate of 13.9 per 1000 in 2019^1^. Since 2008, stillbirth has been defined as the intrauterine death of a fetus after 22 completed weeks of gestation^2^. The declining rate of stillbirth in Sweden has been attributed to advancements in healthcare practices, especially the management of post-term pregnancies exceeding 41 weeks of gestation^2^. However, despite these changes in healthcare practices, parents who experience an irreversible stillbirth continue to face long-lasting effects on their lives^2,3^.

It is important, but not always possible, for bereaved parents to know the exact cause of stillbirth^4-6^. In Sweden, the primary causes of stillbirth have been identified as placental and/or umbilical cord complications^4^. Recently, a Swedish study reported that women originating from Sub-Saharan Africa face a significantly higher risk of stillbirth^7^. Furthermore, a systematic review found that mothers who experience stillbirth are at risk of developing ischemic heart disease, cerebrovascular disease, circulatory and renal disorders, as well as type 2 diabetes mellitus (T2DM)^8^.

Moreover, previous systematic reviews have confirmed an increased risk of long-term depression, anxiety, stress, and alcohol dependence among women who experience stillbirth^8^, and poorer mental health following stillbirth as symptoms of anxiety, depression, and PTSD^9^. Another systematic review highlighted that stillbirth can lead to devastating psychological, physical, and social consequences, although some parents may also develop resilience, new life skills, and coping capacities^10^. Couples with a history of infertility or no living children before the stillbirth reported experiencing more intense grief^10^.

A previous study emphasized the importance of an interdisciplinary team of professionals trained in stillbirth and perinatal loss, to help prevent negative emotional consequences for parents^11^. A Taiwanese prospective follow-up study identified key risk factors one year after stillbirth, including the mother, having no previous children, and receiving low levels of socioemotional support from the husband’s parents^12^. In addition, a recent study on subsequent pregnancies after stillbirth concluded that this period is extremely stressful and requires patient-centered care and support, both physically and psychologically^13^.

Longitudinal studies on stillbirth are scarce^14^, and there is a lack of research focusing on parents’ memories, feelings, reflections and insights one year after experiencing stillbirth. Recent Swedish studies have shown that parents face profound sadness, pain, and grief one month after stillbirth^5^, but reported feeling a glimmer of light at the end of the tunnel six months later^6^. These studies were based on data collected from participants who also contributed to the present study one year after the stillbirth.

This study aimed to further explore and deepen the understanding of parents’ memories, perspectives, reflections, and insights regarding the grieving and recovery process over time. Additionally, the study sought to investigate parents’ mental and reproductive health, their desired healthcare support, and their quality of life up to one year after the stillbirth.

## METHODS

### Design

A mixed-methods design was used, with individual in-depth interviews and quantitative statements about parents’ experiences of stillbirth. The quantitative results are descriptive and the study is convergent^15^. The study included nine parents who had experienced a stillbirth one year earlier. It constitutes the third part of a longitudinal study, which follows each participant over two years, and includes parents who experienced stillbirth after 22 completed weeks of gestation.

### Sample

The purposive sample consisted of bereaved parents who had experienced a stillbirth after 22 completed weeks of gestation, with interviews conducted one year after the event. These parents had previously participated in the initial study one month after the stillbirth, with a follow-up six months later. They were interviewed again at a second follow-up one year after the stillbirth.

### Recruitment

All managers of hospitals with labor wards in Sweden, along with the Swedish Infant Death Foundation, were contacted and invited to assist in recruiting parents who had experienced a stillbirth. Of the 38 clinics approached, 10 responded positively, expressing their willingness and interest to participate. Ultimately, nine agreed to participate, affirmed their interest, and signed a written consent form.

### Data collection

Nine individual in-depth interviews in Swedish were conducted via mobile phone by the first author between October 2022 and July 2024, one year after the stillbirth. Data were collected over a relatively extended period of time.

The duration of each in-depth interview ranged from 65 to 298 minutes (mean=32.4 minutes) for a total of approximately 19.52 hours. All interviews were audio-recorded with the participants’ permission. Before each interview, the interviewer confirmed that the participant was alone in a quiet, disturbance-free room. Interviews followed a semi-structured interview guide, with an initial question: ‘Please, would you like to share the first thoughts that come to your mind, one year later, when you were told at the hospital that your baby had died in the womb?’. All interviews were transcribed verbatim before data analysis. Data saturation^16^ was achieved after eight interviews, when no new information emerged.

Thereafter, participants were asked to rate and reflect on eleven statements previously developed in a study of stillbirth^5^, relating to various aspects of their experience. These included: parents’ assessment of knowing the reason for the stillbirth; memories of holding the baby in their arms; memories of holding the baby close and caring for the deceased baby during the postpartum period*;* assessment of knowing the reason for the stillbirth now and in the future; thoughts and longings for their stillborn baby; quality of life; mental health; needs for professional support (midwife, psychologist, counsellor); relationship with their partner; ability to work; and the importance of work. The statements were assessed using 3–4-point Likert scales.

### Theoretical framework

The study was based on the theory of the Dual Process Model^17,18^ which highlights grief as oscillating between loss-oriented and restoration-oriented coping, capturing temporal variation in experiences. Guided by this framework, a qualitative design was used, with semi-structured interviews exploring parental coping, meaning-making, and prolonged grief following stillbirth.

### Ethical considerations

The Regional Ethics Committee (Dnr: 2021-06536-01) approved this study, and ethical considerations were upheld throughout. Each participant received oral and written information about the longitudinal study and signed an informed consent form. Participation was voluntary, and participants had the possibility to ask questions before and after their in-depth interviews. If the interviews raised any thoughts or concerns, participants were offered the opportunity to contact the researcher responsible for the study. Furthermore, advice and, if needed, referrals to appropriate professional support services were provided.

### Analysis

In this study, the statements were analyzed using descriptive statistics. A thematic network analysis was performed to systematize, organize, and describe the study results. Interpretation was employed during this analysis to identify abstract themes^19^. Thereafter, each author independently analyzed the data inductively, according to the thematic network analysis model of Attride-Stirling^19^. Furthermore, the authors individually read the data several times to gain an overall understanding of the whole content. Meaningful text segments, such as sentences, were then organized into the three levels: basic themes were merged to shape organizing themes, which were subsequently grouped into a global theme. Lastly, patterns were interpreted and illustrated with verbatim quotes. In the Results section, the interview number, gender, parity, and age of the participants are presented. Convergent analysis includes collecting quantitative and qualitative data simultaneously, analyzing them separately, and then integrating them to provide a comprehensive understanding of the research problem. Both authors convened to discuss and revise all analytical decisions until consensual validation was reached (see Table 1 for the analytical scheme). Results of the eleven statements are presented with numbers in Table 2.

**Table 1:** Example of quotes, basic themes, organizing themes and global theme in thematic analysis of a longitudinal study based on interviews with nine parents with stillbirth one year earlier in Sweden, 2022–2024

*Quote*	*Basic theme*	*Organizing theme*	*Global theme*
*‘In retrospect a terrible and extremely tragic message – a real nightmare.’*	Parents’ etched memories and reflections of the suddenly interrupted and complicated pregnancy		
		Impossible to close the heavy door to indelible memories of the stillborn baby	
*‘I knew it was more definitive and that I had to bid farewell after birth, but I’m happy that I could carry the baby girl for four more days.’*	Unwavering memories of carrying, giving birth and staying in hospital after birth		
			Despite a sudden tragic and life-changing stillbirth, the recovery progress protects the existentially deep wound with a paper-thin membrane
*‘I lowered him down in the grave myself and I go there almost daily as it is important for me to visit him.’*	Contributing factors in the recovery phase		
		The balance of personal and social factors and internal processes enables increased quality of life	
*‘When I haven’t felt fetal movements for a while, I get worried and want immediately check-ups.’*	Parents’ reproductive health and the required healthcare support during subsequent pregnancy		

**Table 2:** Overview of the quantitative statements assessed by nine parents who experienced stillbirth one year earlier in Sweden, 2022–2024 (N=9)

	***Completely***	***Partly***	***Not at all***	
Parents’ assessment of knowing the reason of stillbirth	4	2	3	
	***Very good***	***Good***	***Partly good***	***Not good***
Parents’ memories of holding the stillborn baby in their arms	8	0	1	0
Parents’ assessment of their mental health	2	2	3	2
Parents’ assessment of their ability to work	2	2	3	2
Parents’ assessment of their relationship with their partner	9	0	0	0
Parents’ assessment of their quality of life	2	6	1	0
	***Very important***	***Important***	***Partly important***	***Not important***
Parents’ memories of holding the baby close and caring for the dead baby during the postpartum period	9	0	0	0
Parents’ assessment of knowing the reason of stillbirth now and for the future	8	0	1	0
Parents’ assessment of the importance of their work	4	5	0	0
	***Very often***	***Often***	***Seldom***	***Not at all***
Parents’ assessment of thinking and longing for their stillborn baby	6	2	1	0
Parents’ assessment of their need of professional support (midwife, psychologist, counsellor)	3	1	5	0

The authors are midwives and usually involve caring for families with live-born babies. Despite this, the authors have experience of caring for families with stillbirth, which enabled them to approach the parents during the interviews. Furthermore, the authors adopted a clear outsider’s perspective in the analysis.

## RESULTS

### Background of the participants

The participants comprised six women and three men, ranging from 28 to 54 years of age, with a median age of 33.0 years. Their marital statuses were either cohabiting with a partner (n=3) or married (n=6). The pregnancies ranged from 22 to 39 completed weeks of gestation (mean=34.4 weeks), and four informants had undergone *in vitro* fertilization (IVF) before the stillbirth. Six participants had no previous children. Altogether, they reported subsequent pregnancies, births, and miscarriages (n=9), which included four ongoing pregnancies, two live births, and three miscarriages at follow-up at one year. Seven participants worked full-time or less, one was on maternity leave, and another was on sick leave. Participants resided in different geographical locations across Sweden. One was living in a town, and eight were in the countryside as follows: southern Sweden (n=3), middle Sweden (n=4), and northern Sweden (n=2).

### Thematic network

**Figure 1:**
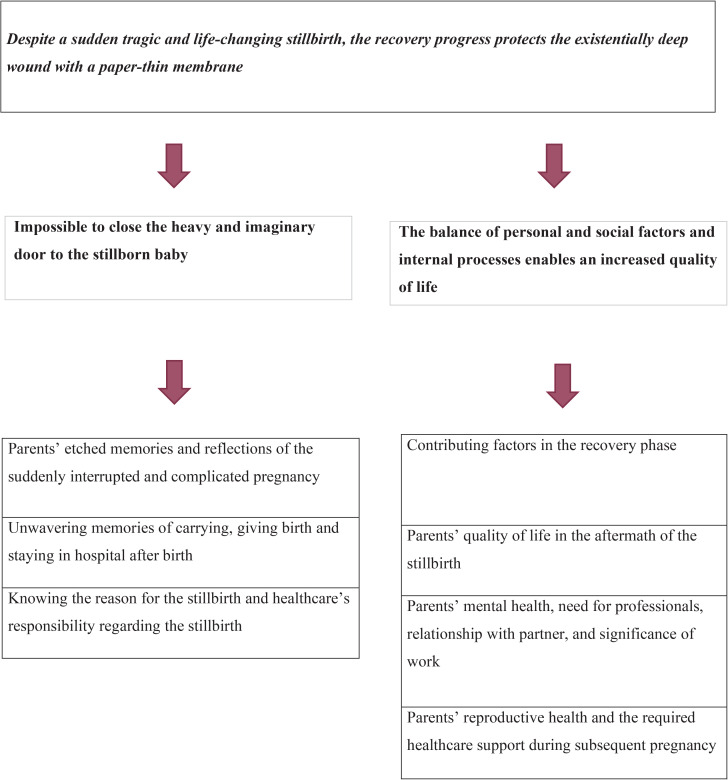
Network of global, organization and basic themes describing parents’ memories and reflections of stillbirth and increased quality of life one year after stillbirth

A thematic network was created, with the global theme: ‘Despite a sudden, tragic, and life-changing stillbirth, the recovery progress protects the existentially deep wound with a paper-thin membrane’. The global theme consisted of two organizing themes: ‘Impossible to close the heavy door to indelible memories of the stillborn baby’ and ‘The balance of personal and social factors and internal processes enables an increased quality of life’ (Figure 1).

The global theme reflected parents’ enduring, tragic memories and insights of the previous stillbirth, and an increasingly conscious strengthening of their ability to move forward in daily life. The recovery process had progressed to different levels among parents, regardless of gender and age. It included a high level of talking and sharing, especially with their partners and, at times, others, as well as thinking about their beloved stillborn baby. Mental health remained poor or worsened for some parents compared with six months after the stillbirth. Despite this, quality of life was generally assessed as good or very good. Overall, an existentially deep wound persisted in the parents’ souls and hearts after the stillbirth, but it was covered by a paper-thin membrane.

The organizing theme ‘Impossible to close the heavy door to indelible memories of the stillborn baby’ comprised three basic themes: 1) ‘Parents’ etched memories and reflections of the suddenly interrupted and complicated pregnancy’; 2) ‘Unwavering memories of carrying, giving birth and staying in hospital after birth’; and 3) ‘Knowing the reason for the stillbirth and healthcare’s responsibility regarding the stillbirth’.

### Parents’ etched memories and reflections of the suddenly interrupted and complicated pregnancy

Parents could clearly remember the deep and irreversible news they received at the hospital regarding the stillbirth, even one year later. They continued to experience deep sorrow and emptiness, finding it impossible to understand, and surreal to believe. One male participant described the sad message as something he could still remember clearly, a message that never changed, and that belonged to the same deep sorrow and sense of loss one year later. Another male participant remembered the unbelievable news:

*‘I couldn’t believe that this had happened to me (us), and I was destroyed and could hardly speak. My wife’s face was in a shock phase - she became silent and didn’t react at all as she was psychologically not there.’* (Interview 9, man, 1 child, 40 years)

Two women described the horrible message in different ways. The first one thought about the emptiness the message created, describing it as a huge abyss, and the incomprehension that the baby had died, alongside worries about how she should manage in the future, altogether causing a total free fall in her life. The second woman stated:

*‘It was a hard time there and then, but nowadays, I have accepted this and learned to deal with the grief, allowed myself to be sad if I need to, and I have landed inside myself in a completely different way.’* (Interview 3, woman, 2-gravida/1-para, 32 years)

All parents responded to a question about whether they would have acted differently when fetal movements became absent or diminished, or when other significant complications arose during the pregnancy. This question became essential and relevant and was reflected upon in relation to the stillbirth that had occurred one year earlier. Four parents stated that they would have acted differently when complications occurred during the pregnancy. This included seeking medical care earlier and having a more critical attitude towards healthcare professionals when significant health issues arose. Four parents affirmed their stance of not having acted differently, even one year after the stillbirth.

### Unwavering memories of carrying, giving birth, and staying in the hospital after birth

Four parents rated carrying the stillborn baby *in utero* to be partly good/good one year after the stillbirth. One woman said:

*‘It was partly good that the stillborn baby was still inside of me, but at the same time I felt scary vibes to have something dead in my womb.’* (Interview 1, woman, 2-gravida/0-para, 28 years)

Another woman reflected:

*‘The thoughts went in other directions, as what is happening and what should I do now, but it was good to have him still in my womb even though he was dead, and there was no inconvenience there and then.’* (Interview 8, woman, 1-para, 37 years)

Other parents expressed negative opinions; one woman described carrying something dead inside her body as feeling very awful and unreal. One man described his reflection, one year after the stillbirth, as an unpleasant and strange feeling, and he wanted the stillborn baby to be delivered immediately so he could quickly leave behind the experience and move on with life. Eight parents assessed their memories of holding the stillborn baby in their arms as very good. One woman expressed regret that she had not initially held the stillborn baby in her arms when the baby girl was still warm and soft. All nine parents assessed the memories of holding the baby close and caring for the deceased baby during the postpartum period as very important.

Seven parents experienced vaginal births, and six of them considered this mode of birth as good/very good. They highlighted that vaginal birth was the most physically optimal for the body and facilitated the psychological processing of the time, allowing them to gradually come to terms with the irreversible stillbirth. They expressed important feelings and understanding about parenting a small family. One woman declared that she had experienced two previous non-traumatic births; this third one went very well and quickly, with good pain relief, but she felt less awareness and self-control, which she largely mourned later. One woman described her vaginal birth as allowing her to remain close to the stillborn baby throughout. Another woman emphasized that a vaginal birth posed no danger to her own health.

### Knowing the reason for the stillbirth and healthcare’s responsibility regarding the stillbirth

Six parents reported that they knew the reason for the stillbirth either partly/completely one year after the event. One man explained that he received the final explanation immediately upon admission to the hospital, which he appreciated both initially and later on. One woman stated that she had been aware early in the pregnancy that her baby had a heart complication, which could lead to later stillbirth. Eight parents assessed knowing the reason for the stillbirth, both now and for the future, as important/very important. One man expressed:

*‘Today, it is super important for me to know the reason before a future pregnancy occurs – if it depends on me, my wife, or the baby’s health.’* (Interview 2, man, expecting second baby, 33 years)

Similarly, one woman stated:

*‘I feel angry all the time because I want to know why my baby died in my womb since I don’t smoke, drink, or take drugs. But am I missing something else? I’m brooding and blaming myself all the time.’* (Interview 4, woman, 1-para, 32 years)

Six parents held healthcare professionals partly responsible for the stillbirth, citing missed check-ups and a failure to induce labor after an abnormal ultrasound examination. Another woman expressed strong concern that she was not taken seriously when her baby was diagnosed as small for gestational age (SGA) and did not achieve the estimated pregnancy weight.

The organizing theme, ‘The balance of personal and social factors and internal processes enables an increased quality of life’*,* comprised four basic themes: 1) ‘Contributing factors in the recovery phase; 2) ‘Parents’ quality of life in the aftermath of the stillbirth; 3) Parents’ mental health, need for professionals, relationship with partner, and significance of working’; and 4) ‘Parents’ reproductive health and the required healthcare support during subsequent pregnancy’.

### Contributing factors in the recovery process

Eight parents reported thinking about and longing for their stillborn baby often/very often (daily to weekly). One of them clarified:

*‘I think several times daily of the stillborn baby, but nowadays, it is with less acute grief.’* (Interview 6, man, no child, 54 years)

A woman said that she thought about her stillborn baby daily, but at this stage, not every minute. All participants described what they mostly thought about regarding the loss one year after the stillbirth. Three clear categories emerged: reflections on the characteristics of lost and growing baby, wishes for the baby to be here and now, and recollections of the initial memories of the stillborn baby at the hospital. Seven parents looked at photographs of the stillborn baby often/very often. Some parents regretted not having taken more photos of the stillborn baby, which they felt could have increased their understanding and healing.

Five parents described thinking about and longing for their stillborn baby as partly difficult/difficult one year after the loss. These expressions included strong feelings of sadness and emptiness that could not be ignored and were linked to the high expectations they had held during pregnancy and before the stillbirth. Parents also described navigating the many deep emotional labyrinths and experiencing several daily ups and downs. They dealt with their deep thoughts in different ways, including feeling sad, crying, and talking with partners and others, as well as psychologists, counsellors, and deacons. One woman engaged in writing down her feelings and thoughts at the moment, which allowed her to feel a sense of calm. She stated:

*‘When I think of my stillborn baby, I could handle it well, and such thoughts often lead to something good and creative inside. But when I feel anxiety, it’s good for me to write things down on paper, and often I cry simultaneously.’* (Interview 5, woman, 0-para, 40 years)

Other parents sent loving thoughts to their missed and beloved baby and tried to understand that nothing more could have been done during the tragic event. Additionally, they recalled the good memories and moments they had with the stillborn baby postpartum and reflected on the beauty they could find amid the tragedy.

Eight parents talked about the stillborn baby with their partners often/very often, one year after the stillbirth. It included daily conversations and fewer frequent discussions than before. All participants described various topics of discussion from past to present reflections. Past reflections included how the stillborn baby had looked and the softness experienced at birth and postpartum. Present reflections included fantasies of the stillborn baby as a living toddler, with various characteristics at one year of age. One participant added that conversations varied; at one moment, at the grave, they had spoken to the stillborn baby about soon becoming a big brother. Despite this, one woman expressed sadness that no one in her nuclear family or relatives talked about the stillborn baby anymore.

Five parents visited the baby’s grave often/very often, and six parents considered these visits as important/very important. The frequency of visits varied widely, ranging from twice daily to three times during the first year. Some parents emphasized the importance of the grave as a place to breathe, talk to the stillborn baby, and honor the baby with candles and flower decorations. The grave was described as the ultimate resting place for the physical body. Others, who visited the grave less frequently, explained that the grave caused great anguish, and instead created a special corner at home with all kinds of visible memories to commemorate the baby.

### Parents’ quality of life in the aftermath of the stillbirth

Eight parents rated their quality of life as good/very good. They reported different improvements in their current lives, including less intense feelings than before, appreciation of the time spent with friends, and the reflective perspectives gained over time. Despite this, they explained experiencing feelings of great sadness and loss regarding the baby daily, but they understood that unfortunate things could occur at any time in life beyond one’s control. One participant said:

*‘Nothing turned out as I imagined and the loss of the stillborn baby is great, although I have my wife and an eye on the future.’* (Interview 2, man, expecting second baby, 33 years)

Another participant stated:

*‘I don’t have the life I had before, although I still have similar things to do in the future, but they are not as important as before.’* (Interview 5, woman, 0-para, 40 years)

Almost all participants described the most difficult and painful aspect of their lives one year after the stillbirth as coping with the profound obstacles it created. These included the absence of a complete family and the devastation of plans for an extended family life, which made it hard to learn to live with this tragic event. At the same time, they also reported fewer difficulties and less pain in managing everyday life one year after the stillbirth. Such situations emerged when they felt close to their stillborn baby, supported by memories and mementos at home, and when they experienced great love for their baby. Furthermore, they felt an increasing inner strength and found it easier to talk about their baby without breaking down as before. In addition, experiencing fewer difficulties and less pain was described as a source of gratitude in life, offering the opportunity to recognize the unexpected and genuine love they felt for their baby, and to consider the stillbirth as part of a profound life journey.

### Parents’ mental health, need for professional support, relationship with partner, and significance of work

All parents assessed their mental health one year after the stillbirth. Five informants (3W/2M) described their mental health as partly good/not good. Compared with their mental health at the follow-up at six months, three participants reported the same partly good mental health, whilst another improved from not good to partly good, and the fourth declined from good to partly good. Four parents stated that they often/very often needed professional support (midwife, psychologist, counsellor) after the stillbirth. One participant stated:

*‘I still have great need for support from professionals such as a psychologist, counsellor, and deacon one year after the stillbirth.’* (Interview 6, man, no child, 54 years)

Four participants announced new pregnancies, and another two reported live births. In total, four of them assessed their mental health as good/very good one year after the stillbirth.

Nine parents assessed their relationship with their partner as very good. Reported comments included increased respect, greater appreciation of each other, and feeling stronger than ever. One participant described increased stability and an even stronger relationship one year after the stillbirth. Four parents assessed their ability to work as good/very good one year after the stillbirth, and nine parents considered the importance of their work as being important/very important. Almost all of them worked full-time. They highlighted significant reasons for working, such as possibilities to talk with colleagues and to think about other things while at work. They stated that, while the trauma of the stillbirth was hard and uncertain to overcome, work felt safe and familiar. One participant stated:

*‘It’s good for me to work, and I really need daily routines.’* (Interview 7, woman, 3-gravida/2-para, 30 years)

### Parents’ reproductive health and demand for healthcare support in a subsequent pregnancy

Nine informants disclosed subsequent pregnancies one year after the stillbirth. Four had ongoing pregnancies, two had live births, and three experienced miscarriages. They described feelings of anxiety and worry, but also pride and satisfaction in parenthood. Almost all participants expressed a strong need for reassurance in the event of a subsequent pregnancy. They asked for increased and improved interaction with healthcare services throughout the whole pregnancy and birth. Specifically, they wanted an individualized pregnancy and birth plan developed together with the healthcare professionals, including more frequent medical check-ups such as ultrasounds, cardiotocograph (CTG) controls, and other appropriate examinations. They called for timely and suitable support, empathic responsiveness throughout, and ensuring that both parents and professionals take critical symptoms seriously. One woman stated:

*‘I want much more concrete interaction with the healthcare professionals during this pregnancy, and I think the third trimester will be the most sensitive period. I don’t dare to build up too high expectations that this pregnancy will go well.’* (Interview 4, woman, 1-para, 32 years)

## DISCUSSION

The main findings confirmed an individual, varied, and meaningful recovery process among bereaved parents one year after the stillbirth. The parents maintained a sense of closeness to and significance of their stillborn baby, despite ongoing existential ups and downs. They recalled beautiful emotional scenarios with their beloved baby, but also experienced the enduring pain of loss and absence. Although waves of grief persisted, they were generally not as deep and frequent as before, and parents managed them with increasing resilience in the recovery process. Over time, the recovery phase added greater quality of life, in favor of new pregnancies and live births, and significance of their working ability, mostly full-time. The participants generously shared their unwavering and tragic memories, along with their assessments of thoughts, perspectives, and reflections, as well as quality of life, and mental and reproductive health, one year after the stillbirth. The in-depth interviews flowed naturally and voluntarily, and the participants expressed great interest in reflecting on the profound significance of the irreversible, abrupt, and complicated ending of their pregnancies. The recovery process is consistent with previous studies which affirm that time contributes to parental healing, and that a recovery continues from a few months to more than one year^12^. There were no differences in the grief reactions in relation to gestational age. The findings align closely with the aims of the study.

The thematic network analysis identified two organizing themes: 1) ‘Impossible to close the heavy and imaginary door to the stillborn baby’, and 2) ‘The balance of personal and social factors and internal processes influences the recovery process’, culminating in one overarching global theme, ‘Despite a sudden tragic and life-changing stillbirth, the recovery progress protects the existentially deep wound with a paper-thin membrane’. This metaphor could be explained as a sudden, tragic, and life-altering stillbirth, and the process of recovery appears to shield the profoundly existential wound with a fragile, almost imperceptible protective layer, akin to a paper-thin membrane.

The organizing themes were identified from the exploration of enduring tragic memories of the stillbirth, as well as reflections on ongoing processes in recovery. The recovery process was regarded as significant, with overall and daily quality of life taking a central role.

In the organizing theme, ‘Impossible to close the heavy and imaginary door to the stillborn baby’, the results unveiled that ‘Parents’ etched memories and reflections of the suddenly interrupted and complicated pregnancy’ illustrated how unpleasant and shocking memories and experiences were still related to the fatal outcome one year after the stillbirth. Previous studies have confirmed such reactions and behaviours^5,6,20^, as well as the existence of strong bonds between the living and the dead, with memorialization recognized as an individual choice for the bereaved^21^. Furthermore, this basic theme also highlighted the need for professionals to provide more comprehensive and appropriate medical information to parents during pregnancy, which could prevent fatal outcomes. At the same time, expectant parents may need to adopt a more questioning and critical stance towards professional advice. This is in line with a previous systematic review, which highlighted the importance of high-quality bereavement care after stillbirth, along with the development of specific training and service provision to improve support for bereaved parents^22^. Additionally, other studies have shown that women’s experiences of pregnancy complications emphasize the need for effective communication from healthcare professionals, which in turn influences their trust in healthcare providers^23,24^.

The results of the basic theme, ‘Unwavering memories of carrying, giving birth, and staying in hospital after birth’ referred to both distressing and positive recollections. Distressing memories included the experience of carrying a stillborn baby *in utero* and giving birth. Positive memories, in contrast, centered on the support provided by professionals during the tragic situation, as well as the opportunity to embrace and care for the stillborn baby in their parental role^5,6^. Additionally, vaginal birth was considered the optimal mode of delivery, due to psychological and physical benefits for subsequent pregnancies and births^3,5,6,25^.

The basic theme, ‘Knowing the reason for the stillbirth and healthcare’s responsibility regarding the stillbirth’ demonstrated that understanding the cause of the stillbirth, whether immediately or eventually, is considered important for the parents in preparing for future pregnancies, which is in line with previous studies^4-6^. Additionally, one study emphasized that it is vital for parents to receive proper counselling on the cause of the stillbirth to be optimally prepared and supported in subsequent pregnancies^26^. Moreover, holding the healthcare professionals partly responsible for the stillbirth has also been reported in previous studies^4-6^.

In the organizing theme, ‘The balance of personal and social factors and internal processes influences the recovery process’, the basic theme ‘Contributing factors in the recovery phase’ reflected important covariation factors that supported recovery, including the passage of time after the stillbirth and the development of new pathways that facilitate the healing process. Recovery involved dealing with memories and experiences concerning the stillborn baby as often as desired and/or needed, either alone, with a partner, or with others. This aligns with a previous systematic review, which noted a lack of intervention research for women after stillbirth, especially during the inter-conception period as part of stillbirth aftercare^27^.

The basic theme, ‘Parents’ quality of life in the aftermath of stillbirth’ showed a gradual brightening of everyday life. Parents gained a clear insight that tragic outcomes in life cannot be fully controlled and, thus, learned to live as realistically as possible. A new sense of pleasure and joy in daily life, accompanied by a stronger capacity to avoid thinking about and dwelling on sad memories, was their first reaction one year after the stillbirth. A Swedish study similarly found that bereaved parents with a previous stillbirth could engage in activities connected with feelings of joy, especially on anniversaries^21^.

Moreover, the basic theme, ‘Parents’ mental health, need for professionals, relationship with partner, and significance of work’ indicated that mental health was partly poor and, in some cases, worse than at six months post-stillbirth. This is in line with previous systematic reviews, which have shown that stillbirth can have long-term psychological effects^8,9^, although parents may also develop resilience and acquire new life skills and coping capacities^10^.

Additionally, this theme highlighted that paid work remained important/very important one year after the stillbirth. However, a previous international study reported that bereaved parents often return to work out of financial necessity rather than a genuine readiness to rejoin the workforce^28^.

The basic theme, ‘Parents’ reproductive health and required healthcare support in a subsequent pregnancy’ demonstrated that new pregnancies necessitated increased interactive support from healthcare professionals to ensure a healthy pregnancy, safe childbirth, and a live baby. Professionals should provide respectful, responsive, and reciprocal collaboration with expectant parents, based on improved, evidence-based, and clear information throughout the subsequent pregnancy and childbirth. Nevertheless, a previous UK study identified several examples of good practice but also confirmed a general lack of consistency in providing suitable emotional and psychological support for women during subsequent pregnancies after stillbirth^29^. Other studies have underlined the need for a tailored support system for parents, especially for those who felt they lacked opportunities to grieve^30^, and the importance of providing thoughtful, empathetic, and team-based care in all subsequent pregnancies following a stillbirth^5,6,13,31.^

### Strengths and limitations

The study has several strengths and limitations. Key limitations include potential recall bias, as data were collected one year after the stillbirth, and selection bias, since participants were highly motivated volunteers. The potential influence of intervening events during this interval cannot be excluded and may have affected the results. Only nine individuals participated in the study, which is a limitation *per se,* and data saturation was achieved after eight in-depth interviews, as no new information emerged. Longitudinal studies focusing on stillbirth are challenging, particularly due to the difficulty of recruiting bereaved parents. Furthermore, future longitudinal studies are needed to explore bereaved parents who develop prolonged or profound mental health problems^14^. Although data saturation was reached, there is no guarantee that recruiting more participants would not have provided new insights or added more nuances to the results.

The study used mobile phones for data collection, which may have facilitated participation, providing a more private and less stressful environment for the participants. However, the lack of visual observation of participants’ body language may have resulted in missed information. The authors’ pre-understanding and previous experiences in this field may also have influenced the research process and/or the outcomes. As the authors were midwives, discussions during the analysis were informed by their previous understanding of the subject^19^.

Trustworthiness was ensured through detailed documentation of the analysis and evaluation of thematic similarities and differences, dependability being one way to ensure rigour^32^. Both authors are experienced midwives and familiar with qualitative methods, which ensured the trustworthiness of the translation. In order to maintain the integrity of the data, a special focus was placed on the citations. The authors performed the steps in the analysis process individually and together, which ensured credibility and confirmability. By providing detailed descriptions of the content and context of the interviews, the selection and characteristics of the participants, data collection, and lastly the analysis process further supported the transferability of the study.

Furthermore, the sociodemographic and ethnic backgrounds of the participants were described in detail. All parents had taken part in previous studies performed one month and six months after the stillbirth, respectively. The present study followed the same parents up to one year after the stillbirth. The first author conducted all the in-depth interviews and used the same interview guide. All the rules for performing in-depth interviews and analyzing data were carefully followed. The participants’ quotes validated the basic themes. Furthermore, both authors collaborated in data collection, analysis, and interpretation of the results. Another strength is the combination of qualitative and quantitative data, which contributed to the robustness of the findings. The eleven quantitative statements will be used to describe trends over time, but will not be used for generalization, due to the limited number of participants.

## CONCLUSIONS

The parents described an increased stability and strength in coping with the loss of their stillborn baby one year after the stillbirth. Nevertheless, they still carried unwavering, tragic memories and longings for their deceased baby. The parents emphasized that, in the event of subsequent pregnancies and births, they require clear, evidence-based, and interactive information, as well as serious responsiveness and respectful treatment from healthcare professionals, taking into account their previous stillbirth.

## Data Availability

The data supporting this research cannot be made available for privacy or other reasons.
